# Health Education about Rheumatic Heart Disease: A Community-Based Cluster Randomized Trial: Rheumatic Heart Disease Educational Strategies

**DOI:** 10.5334/gh.347

**Published:** 2020-06-17

**Authors:** Kaciane K. B. Oliveira, Bruno R. Nascimento, Andrea Z. Beaton, Maria Carmo P. Nunes, José Luiz P. Silva, Lara C Rabelo, Marcia M. Barbosa, Cássio M. Oliveira, Mariana D. Mata, Waydder Antônio A. Costa, Augusto F. Pereira, Craig A. Sable, Antonio L. P. Ribeiro, On behalf of the PROVAR (Programa de RastreamentO da VAlvopatia Reumática e outras Doenças Cardiovasculares) investigators

**Affiliations:** 1 Serviço de Cardiologia e Cirurgia Cardiovascular e Centro de Telessaúde do Hospital das Clínicas da UFMG, Belo Horizonte – MG, BR; 2 Departamento de Clínica Médica, Faculdade de Medicina da Universidade Federal de Minas Gerais, Belo Horizonte – MG, BR; 3 Cardiology, Children’s National Health System, Washington – DC, US; 4 The Heart Institute, Cincinnati Childrens Hospital Medical Center, Cincinnati – OH, US; 5 University of Cincinnati Medical School, Cincinnati, OH, US

**Keywords:** rheumatic heart disease, health education, worked examples, schools, screening

## Abstract

**Introduction:**

The burden of rheumatic heart disease (RHD) is still high in Brazil. Lack of population awareness about the disease limits the efficacy of prevention programs. We aimed to evaluate the effectiveness of education on RHD in schools, comparing the conventional expository teaching method with tablet-based worked examples.

**Method:**

A prospective, cluster randomized trial was conducted over eight months in six randomly selected low-income Brazilian public schools. Each class was considered a cluster (total: 90), being randomized 1:1 to receive one of the educational methods. Pre-test evaluated students’ prior knowledge on RHD. Post-tests, 10 days, and three months later, evaluated retention of knowledge.

**Results:**

At total 1,301 students (52% female) completed the study, being 63% from high school. Baseline knowledge about RHD was universally low (average score expository classes [G1] 33.9% vs. worked examples [G2] 32.5%, p = 0.23). A significant but similar improvement was observed in both groups in the immediate post-test (pre- vs. post: p < 0.001): G1 57.5% vs. G2 56.7%, p = 0.69. In the late post-test, a significant 20% worsening was observed in both groups and the final scores were again similar: G1 45.0% vs. G2 45.9%, p = 0.87. Highschool students had higher scores (p < 0.001), and girls had better overall performances than boys (p < 0.001).

**Conclusion:**

The novel technology of tablet-based worked examples had similar results compared with expository classes for RHD education in schools. Both educational processes resulted in modest gains in knowledge, with low retention. More studies are needed to determine if increased knowledge leads to behavioral changes that could reduce RHD burden.

**Highlights:**

- In a cluster-randomized trial, two different educational strategies about Rheumatic Heart Disease (RHD) for children – standard expository classes and worked examples based on interactive modules in tablet computers – were compared in public schools of underserved Brazilian neighborhoods.
- Baseline knowledge was low, and the novel tablet-based technology had similar results compared with traditional teaching for RHD education in schools, with no differences in acquisition and mid-term retention of knowledge.
- Both educational interventions resulted in similar 71% improvement in the immediate post-test, although with suboptimal retention, with over 20% worsening in three months in both groups.
- Our data suggests that the optimal strategy for RHD education is yet to be determined, and future studies should be warranted to determine if increased knowledge leads to behavioral changes that could reduce disease burden in endemic areas.

## Introduction

Rheumatic heart disease (RHD) is the main acquired cause of cardiovascular morbidity and mortality in children and young adults, especially in poor regions of the globe [1,2], causing significant impact on health systems due its late sequelae, especially advanced carditis [3]. It is strongly associated with restricted access to healthcare, overcrowding and nutritional deficiencies [4,5], markedly affecting underserved populations of low- and middle-income countries [1,6].

Being a preventable disease, its primary prophylaxis consists of simply recognizing and treating–at the primary care level–pharyngitis caused by group A beta-hemolytic Streptococcus (GAS), with widely available antibiotic regimens [6,7]. Once an episode of acute rheumatic fever (ARF) occurs, or in the presence of RHD findings, secondary prophylaxis consists of Benzathine Penicillin (BPG) injections every 3–4 weeks, preventing recurrent infections and eradicating the causative agent, thereby reducing the risk of worsening cardiac involvement [1,8,9,10].

Population’s knowledge about prevention, prophylaxis and long-term consequences of ARF and RHD is extremely limited. Therefore, educational strategies focusing on such topics [11,12] may be inexpensive and effective ways to reduce RHD burden in the long run [13,14]. Health education processes carried out in schools are effective in improving students’ knowledge about diseases, and their effectiveness can be assessed through structured tests applied before and after interventions [15]. In this setting, the evaluation of novel technologies should be warranted to overcome limitations of the traditional education models, focusing the teaching-learning process on the student and developing abilities such as autonomy, which may favor rethinking daily practices [16].

Different learning theories have been proposed, such as the meaningful approach, which emphasizes learning as an inductive process, starting from a primary understanding of general concepts, until the understanding of specific details. It suggests expository teaching as the method of choice, with mandatory interactions between educators and students [17]. It’s useful for teaching the relationship between concepts or for introducing unknown or difficult topics to the learner’s cognitive structure. Conversely, the theory of cognitive load suggests the superiority of learning from examples, presupposing that it promotes the construction of schemes which can be used to solve problems related to the concepts studied. This process theoretically involves 3 steps: first, concepts and principles of a domain are introduced to the students; second, they work examples that are instances of these concepts and principles; third, problems which require the application of the principles learned are solved [18]. However, the ideal methodology for health education is yet to be determined.

We aimed to evaluate the effectiveness of transmission and retention of knowledge resulting from an educational process on pharyngitis, ARF and RHD in Brazilian public schools, comparing the results of two teaching methods: the conventional method, using expository classes with slide presentation, and the experimental method, with an example-based learning strategy utilizing worked examples provided in interactive tablet-based modules.

## Methods

### Setting and participants

The data analytic methods, and study materials will be made available to other researchers for purposes of reproducing the results or replicating the procedures, from the corresponding author upon reasonable request. The PROVAR (*Programa de RastreamentO da VAlvopatia Reumática*) study takes place in the state of Minas Gerais, southeast Brazil, being conducted in the metropolitan areas of Belo Horizonte (the capitol, 4.9 million inhabitants) and Montes Claros (north of the state, 0.58 million inhabitants), under the auspices of the Universidade Federal de Minas Gerais and the Telehealth Network of Minas Gerais [19], in collaboration with the Children’s National Health System, Washington-DC, USA. Ethics approval was obtained from the institutional review boards of the participant institutions and from the state and city Boards of Health and Education. The methodology of PROVAR has been described elsewhere [20,21]: summarily, the study utilizes non-experts for image acquisition, on portable and handheld devices for RHD echocardiographic screening, and telemedicine interpretation by experts in Brazil and the US, according to the 2012 WHF [8] criteria. All screening activities are preceded by an educational curriculum on ARF and RHD.

This study is a community-based cluster randomized trial with quantitative, longitudinal, and prospective data collection. Students from six public state schools located in underserved areas of Belo Horizonte, in elementary (grades 6–9) and high school (grades 1–3) were enrolled during the second semester of 2016 and first semester of 2017. The State Board of Education selected the schools based on socioeconomic indexes and local priorities.

### Study procedures

Initially, a prospective pilot study was conducted in 5 public state schools (3,200 schoolchildren) of Belo Horizonte metropolitan area, from September 2014 to March 2015, with the conventional educational process (expository classes). A structured questionnaire containing 15 multiple-choice questions was developed in collaboration with investigators from the Ugandan RHD program, considering different domains of knowledge about pharyngitis, ARF and RHD (definitions, recognition, prevention and treatment). A pre-test was applied to students from the sixth year of elementary school to the third year of high school, prior to any educational process, to evaluate the baseline knowledge. The conventional expository curriculum was then delivered and 1,025 students underwent an immediate post-test (10 days after the intervention) with the same questionnaire. Results showed a median 20% improvement comparing pre-and post-tests in all age groups [22]. Based on this pilot, suggesting a moderate retention of knowledge, we opted to use the same questionnaire in the present cluster randomized study.

Initially, additional schools selected by the State Board of Education were visited and study procedures were explained to directors and representatives, and the trial initiation was scheduled. In the first contact with the students, before education processes were conducted, the structured questionnaire was applied to assess the prior knowledge about pharyngitis, ARF and RHD (pre-test). Students were also asked to answer a general demographic and socioeconomic questionnaire containing age, sex and measures of family income. After that, randomization by cluster (classes) was carried out, according to educational level (6^th^ to 9^th^ years of elementary school and 1^st^ to 3^rd^ year of high-school), in a 1:1 fashion, between G1: conventional (expository classes) and G2 experimental (tablet-based worked example) health education processes.

Subsequently, conventional (expository classes with structured slide presentation, taught by the same research nurse who conducted the pilot study) and experimental (worked examples provided in individual interactive modules for mobile tablet devices) health education interventions were applied. Both processes lasted approximately 20 minutes and contained the same aforementioned topics on pharyngitis, ARF and RHD, with appropriate language for the age groups involved.

Ten days (immediate post-test) and three months (late post-test) after the intervention the same standardized questionnaires were applied to assess students’ retention of knowledge and effectiveness of the educational strategies. Students had no access to the correct choices nor to previous answers or grades between tests. These were presented and discussed after the late post-test, and educational flipcharts containing information about RHD for children and families were distributed. Questions left unmarked were considered to be wrong.

### Statistical analysis

A longitudinal comparison between the two of the educational processes (interventions) was carried out at the three time points (pre-test, immediate post-test and late post-test), stratified by education level (elementary school and high school), sex and family income, in order to evaluate acquisition and retention of knowledge over time. The pre-specified primary outcome was the number and percentage of correct answers of the conventional (G1) and experimental (G2) groups, at the three time points.

Sample size was calculated based on a median 20% gain in the immediate post-test compared to baseline observed in students undergoing conventional education in the pilot study, and the probability of 30% improvement with the experimental method (50% increase compared to the conventional method). Stratified clusters were defined according to education level (elementary and highschool) and grouped by class, considering a type I error of 5%, 80% power and variation coefficient between groups of 0.25. A sample consisting of 36 clusters was calculated, considering average 30 students per class (N = 1,080). Due to the loss rates, the number of clusters was increased to 90.

Data were entered to the RedCap® online database [23]. The analysis was performed in the statistical software R version 3.4.3, with the utilization of the *foreign* (data reading), *ggplot2* and *gridExtra* (graphics), *geeglm* (regression models), *plyr* (numerical summaries) statistical packages. For the exploratory analysis, data was read in the *data.RData* file, with the insertion of the corresponding 15 correct answer indicators. SPSS version 22 for Mac OSX (IBM Corp., Armonk, NY, US) was used for additional descriptive analyses. Categorical variables, expressed as numbers and percentages, were compared between groups (G1 and G2) using Fisher’s exact test, whereas continuous data, expressed as mean ± SD or median or Q1/Q3 (25%/75%), were compared using Student’s unpaired t-test or the Mann-Whitney U test, as appropriate. A two-tailed significance level of 0.05 was considered.

## Results

**Figure 1:**
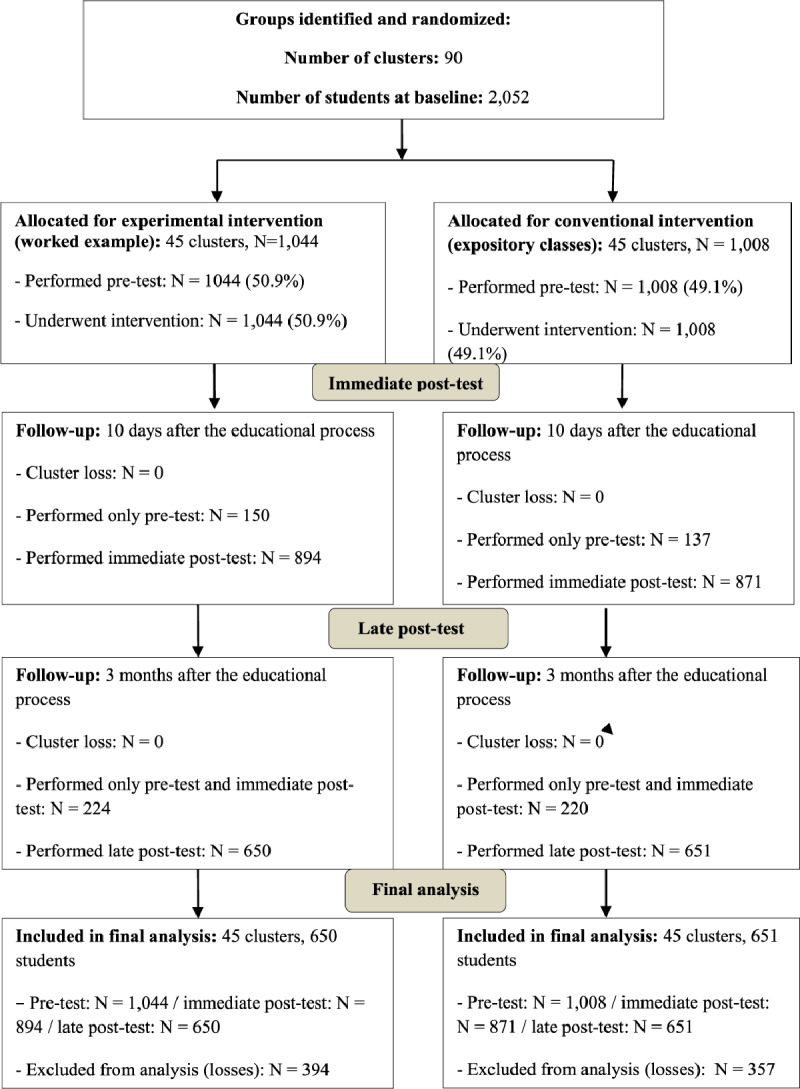
Consort diagram with the total number of participants and losses, number of clusters, monitoring and analysis of study groups.

The baseline sample consisted of 2,052 children and, after losses in the immediate and late post-tests, 1,301 students from 90 classes (clusters) were included in the final per-protocol analysis, being: G1 (conventional), N = 651, 45 clusters and G2 (experimental), N = 650, 45 clusters. Total 478 students (37%) were in elementary school and 823 (63%) in high school (p < 0.001), being 22.8% < 13 years, 26.2% from 13–16 and 51% > 16 years. The study Consort diagram, with detailed information on inclusions and losses is depicted in Figure 1. Participation of girls (677; 52%) and boys (624; 48%) was balanced (p = 0.39), and mean age was similar between groups (G1: 15.2 ± 1.9 vs. G2: 15.1 ± 2.0 years, p = 0.63.

### Loss analysis (patients with incomplete data)

Considering the study setting, there were considerable losses in the post-tests, totaling 751 (37%) students (G1: 357 vs. G2: 394, p = NS). The main reasons were school absence during study days (N = 696), refusal to answer the questionnaires (N = 29) and school dropout or change (N = 26). Table 1 shows the comparison between the students who completed the study protocol and losses throughout the three time points. The number of losses among girls was higher compared to boys, but the rates were similar between groups (65% vs. 62%; p = 0.94). Losses were more frequent among highschool students compared to those in elementary school, and also among participants with lower maternal educational level (lower proportion of complete or incomplete high school).

**Table 1:** Comparison between students who completed the study protocol and losses.

Variable*	Students with complete data (3 time points) (N = 1301)	Losses (n = 751)	p-value

**Sex (female)**	677 (52)	395 (53)	0.807
**Group**			0.651
Expository classes (G1)	651 (50)	357 (48)	
*Worked Example* (G2)	650 (50)	394 (52)	
**Schooling**			<0.001
Elementary school	478 (37)	145 (19)	
Highschool	823 (63)	606 (81)	
**Father educational level†**			0.279
Elementary school (complete or incomplete)	528 (44)	285 (42)	
Highschool (complete or incomplete)	319 (27)	168 (25)	
Superior	88 (7)	61 (9)	
Not informed	256 (22)	163 (24)	
**Mother educational level†**			<0.001
Elementary school (complete or incomplete)	532 (43)	285 (42)	
Highschool (complete or incomplete)	441 (35)	168 (25)	
Superior	87 (7)	61 (9)	
Not informed	186 (15)	163 (24)	
**Father income**‡			0.429
Up to 1 minimum wage	164 (13)	106 (15)	
1–2 minimum wages	220 (18)	133 (19)	
3–4 minimum wages	58 (5)	34 (5)	
≥5 minimum wages	21 (2)	17 (3)	
Not informed	754 (62)	400 (58)	
**Mother income**‡			0.456
Up to 1 minimum wage	335 (27)	189 (27)	
1–2 minimum wages	201 (16)	135 (19)	
3–4 minimum wages	31 (3)	23 (3)	
≥5 minimum wages	13 (1)	9 (1)	
Not informed	661 (53)	361 (50)	

* Values expressed as absolute numbers and percentages for each group. † Father educational level: total answers 1868 (1191 in the group with complete data and 677 in the loss group). Mother educational level: total answers 1923 (1246 in the group with complete data and 677 in the loss group). ‡ Father income: total answers 1907 (1217 in the group with complete data and 690 in the loss group). Mother income: total answers 1958 (1241 in the group with complete data and 717 in the loss group).

### Comparison between groups

**Figure 2:**
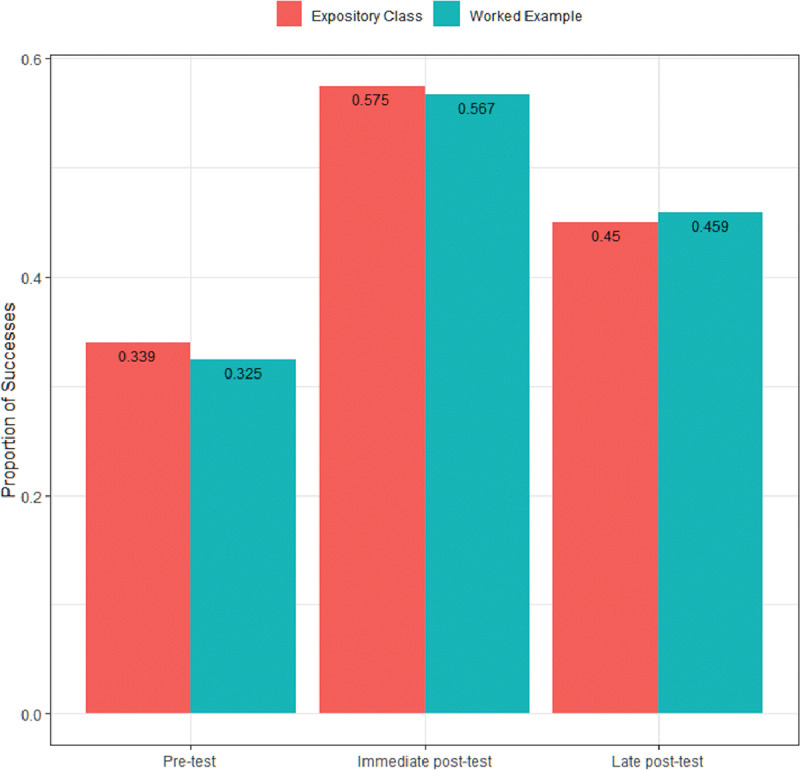
Proportions of correct answers in the pre-test, immediate and late post-tests for the conventional and experimental groups (primary outcome).

**Appendix Figure 1:**
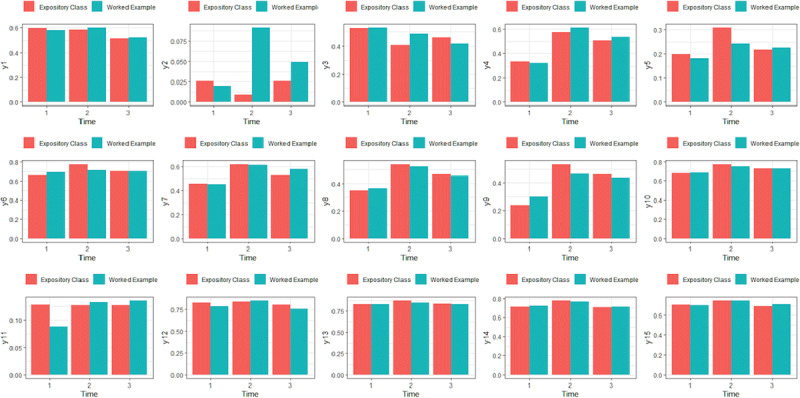
Proportions of correct answers by individual question for the conventional and experimental groups.

Prior to the interventions, general knowledge about ARF and RHD was universally low (mean score G1 34.0% vs. G2 32.3%, p = 0.23). A significant but similar 71% improvement was observed for both groups (expository class and worked examples) in the immediate post-test (pre vs. post: p < 0.001): G1 57.5% vs. G2 56.2%, p = 0.69. For the three month post-test, a significant 20% worsening was observed (p < 0.001), and the final grades were again similar: G1 44.8% vs. G2 45.7%, p = 0.87. Retention rates in relation to immediate post-test were 77.9% and 81.3%, respectively (p = 0.79) (Figure 2). When individual questions were evaluated, the results were relatively homogeneous and again similar between groups in the three tests. Appendix Figure 1 shows the proportions of right answers each of the 15 questions proposed in each time point.

Punctual differences between G1 and G2 were observed in specific questions, although with variable performance in G2. Of note, question two was the most notable outlier for early retention of knowledge, as children undergoing the worked example had significantly better results in the immediate post-test (p < 0.001), although a drop occurred in the late post-test (p = 0.070). Thus, overall results were similar (Appendix Figure 1).

**Figure 3:**
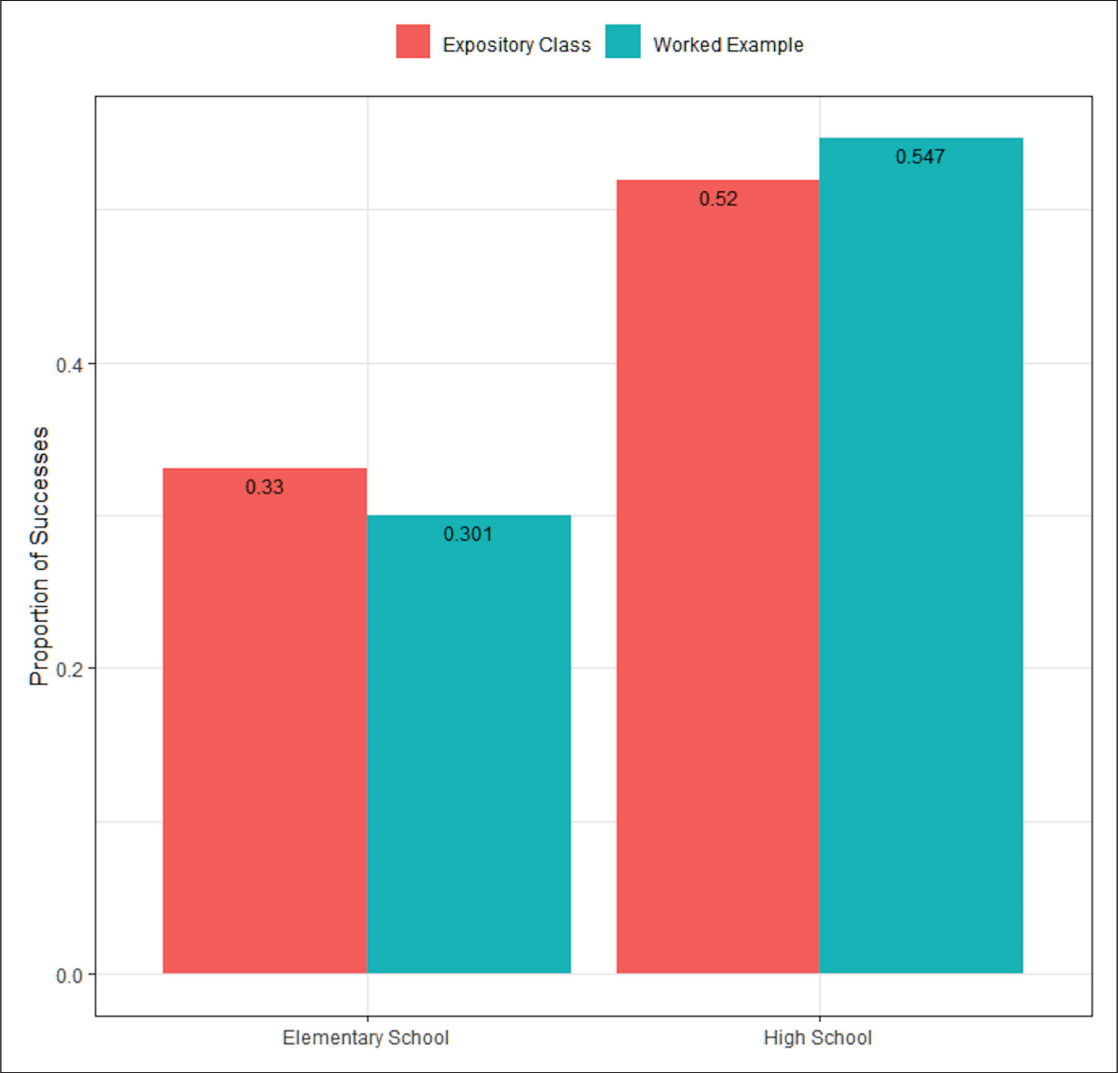
Overall proportions of correct answers by question for children in elementary and high school.

Children in the higher grades (high school) showed higher overall scores in all tests compared to elementary school, with a positive correlation with age (p < 0.001) (Figure 3). Girls also had better overall performances (G1 54.7%, G2 55.6% vs. G1 35.9% G2 32.5%, p < 0.001), but again G1 and G2 were similar. There was no association between parental schooling and performance in the three tests, with similar results in all strata.

## Discussion

Investigating the effectiveness of two different health education approaches on pharyngitis, ARF and RHD in schoolchildren, our study found no differences in acquisition and retention of knowledge comparing expository classes and the novel tablet-based worked examples. Although the groups were similar in performance, we demonstrated that health education processes significantly improve knowledge about the disease, and may play an important role in primary and secondary prevention in the long run [24]. Retention of knowledge, however, was noticeably suboptimal.

Given the potential benefits of population awareness, there has recently been growing interest in the evaluation of novel strategies to deliver health education, for different diseases and in diverse scenarios and focusing on preventable conditions. In a study in the US comparing conventional health education about contraceptive methods carried out by an educator with modules delivered in mobile devices (tablet computers), the findings were similar to our data, with no significant differences between strategies [25]. However, interventions focused on neglected diseases in low-resourced settings have undoubted particularities–associated with education infrastructure and socioeconomic/family background–which require further investigation and personalized approaches.

Marked differences between the three time points are apparent from our data, with a reasonable immediate acquisition of knowledge, contrasting with an over 20% worsening in the late post-test. The significant improvement observed comparing pre and immediate post-tests are in agreement with available data showing that information-based interventions contribute to the overall gain and retention of knowledge about a particular disease [12,15,26]. Conversely, the loss of knowledge over time, denoted by the steep drop in the late post-test, may be associated with the process of ‘mechanical’ learning, possibly due to children being more prone to memorization and less to understanding, resulting in low retention. Thus, both curriculums were probably not meaningful enough, and the lack of subsequent interactions about RHD topics in the classroom between tests may have contributed [27].

Despite the similar results compared to the standard approach, worked examples – especially based on new technology – may be important teaching tools with a potential for better transfer performance and more efficient learning, as previously suggested by studies on cognitive science [28]. Also, they may potentially broaden access to information with the use of technology. On the other hand, the conventional method also had good performance, with significant knowledge improvement from baseline, suggesting that in places with limited access to alternative technological tools, instructional orientation can be optimized to engage students in educational activities [29]. One of the limitations of this approach, however, is the impossibility of delivering health education to large audiences at a time – as it depends on a teacher or supervisor – limiting its practical coverage where personnel is limited. Tablet computers, conversely, could be additionally used for other applications, as house-to-house education in primary care.

The better performance of high-school students compared to those of elementary school may be associated with a greater prior knowledge about health and biology topics, as well as with other previously developed learning skills, allowing for assimilation of higher levels of instructional guidance [30,31,32,33]. In addition, the subjective perception that superior scores among females are associated with greater commitment, attention, motivation and interest during the interventions is supported by previously published studies. Data suggest that the position of boys in terms of educational level, attitudes and behavior is much more unfavorable than that of girls in primary education, and markedly throughout the first phase of secondary school [34].

Schools are a platform for health promotion regarding RHD, since the most vulnerable population–and the ideal target for primordial prevention–are school-age children. Especially in low and middle-income countries, there is a great potential for improvement in access to education, health awareness and dissemination of primary and secondary prevention measures [35]. Besides improvements in students’ knowledge, school interventions conducted by a trained team potentially impact teachers, employees and families, and may contribute for the reduction of RHD burden in the long run [36]. In our study, focus was given to basic concepts, such as the importance of adequately treating pharyngitis, prevention and early treatment of ARF and RHD, considering the low socioeconomic background. In other scenarios, more advanced topics and deeper discussions can be promoted.

Although the traditional classroom will probably be present for years, the incorporation of novel technology-based educational tools must be warranted, considering their potential to reduce costs and add educational value. There are still many lessons to be learned, especially about the fusion of these new technologies with traditional education, given the growing understanding about human learning and the structure of knowledge [27]. Even with neutral results, our study adds to this field highlighting the need for the continuing investigation of teaching technologies for health purposes, as this disease-specific model of tablet-based worked examples did not result in improvement. Addressing local particularities–such as social and cultural issues–for the development of educational tools may lead to better results.

Our study has several limitations. At first, there was a considerable proportion of losses (37% of the sample) noticeably in late post-test. This was associated with several reasons, especially frequent absence and dropout from school, drawbacks of the Brazilian public education. Of note, similar results were observed in studies with similar scope in high-income regions [28], suggesting this may be a general difficulty in carrying out health education in schools. The losses, however, were balanced and did not differ significantly from the final per-protocol sample. Second, the immediate post-test was applied 10 days after education, and better results could have been achieved immediately after the interventions, more accurately reflecting early acquisition of knowledge. However, this additional information about 10-day retention is also valuable for a broad appraisal of the educational processes and short-term information retention. Third, the questionnaires were standardized and not age-specific. Although the age range was short, this may also bias the findings, favoring older students. Even with these limitations, to the best of our knowledge this is the largest trial evaluating new technology for health education on neglected diseases in Latin America, and our findings may help the development and refining of teaching strategies.

## Conclusion

The educational process on RHD in schools has resulted in modest gains in knowledge, with low retention over time. The new technology of worked examples in tablets obtained similar results when compared with expository classes. More studies are needed to determine if increased knowledge leads to behavioral changes that could reduce RHD burden.

## Abbreviations

ARF: Acute Rheumatic Fever

BPG: Benzathine Penicillin G

GAS: Group A streptococcus

PROVAR: Programa de RastreamentO da VAlvopatia Reumática

RHD: Rheumatic Heart Disease

WHF: World Heart Federation
